# Exploring the Properties of Organometallic Lactone-Containing Poly(benzofuran-co-arylacetic Acid): Traditional Synthesis Versus Mechanosynthesis

**DOI:** 10.3390/polym17182511

**Published:** 2025-09-17

**Authors:** Teodora Radu, Alexandrina Nan, Monica Dan, Maria Miclǎuş, Natalia Terenti

**Affiliations:** National Institute for R&D of Isotopic and Molecular Technology, 67-103 Donat Street, 400293 Cluj-Napoca, Romania; teodora.radu@itim-cj.ro (T.R.); monica.dan@itim-cj.ro (M.D.); maria.miclaus@itim-cj.ro (M.M.)

**Keywords:** poly(benzofuran-co-arylacetic acid), 3d transition metal salts, mechanosynthesis, solution synthesis, thermal conductivity

## Abstract

This work describes the synthesis and characterization of novel organometallic polymeric frameworks derived from lactone-based poly(benzofuran-co-arylacetic acid) (PBAAA) ligands complexed with 3d transition metal salts (Co^2+^, Cu^2+^, Zn^2+^). Two distinct synthetic approaches were investigated: conventional solution-based methods and mechanochemical ball milling. A comprehensive spectroscopic evaluation was performed utilizing FTIR, XRD, UV-Vis, and XPS techniques to detail the structural characteristics of the synthesized materials. The thermal assessments were conducted using TGA and thermal conductivity, demonstrating that the chosen synthesis method has a significant impact on the crystallinity, coordination environment, and thermal transport characteristics of the resultant complexes. Remarkably, using the mechanosynthesis, the resulting organometallic polymer materials exhibited enhanced chain ordering and improved thermal conductivity, with a value of 0.32 W/mK, almost double that of the starting polymer. A correlation was identified among thermal conductivity, metal ionic radius, coordination number, and the synthesis method utilized. XPS analysis revealed the presence of multiple oxidation states and varied electronic environments, particularly in copper complexes. These had a direct effect on how they behaved when heated. These results show that mechanochemical synthesis is a useful and long-lasting method to make complex organometallic polymers with thermal properties that can be changed.

## 1. Introduction

The electrical and thermal conductivity of polymeric materials is heavily influenced by the intricate morphology of their polymer chains. Typically, the polymers containing saturated chain structures are materials that are defined as thermal and electrical insulators. This characteristic renders them effective for applications like foam insulation, plastic containers, and electrical wiring [1,2,3,4]. On the other hand, conjugated polymers possess built-in thermal and electrical conductivity due to their large areas of mobile π-electrons and strong intermolecular π-π stacking interactions [5,6]. Polyacetylene (PA) is the first and simplest conducting polymer [7]. Later, considerable improvements in conductivity through the doping procedure led to the granting of a Nobel Prize [8]. Polyaniline (PANI) [9,10], polypyrrole (PPy) [11,12], polythiophene (PTH) [13,14], poly(para-phenylene) (PPP) [15], poly(-phenylenevinylene) (PPV) [16], and polyfuran (PF) [17,18] are other notable conducting polymers. All of these show improvements in conductivity when they are protonated (oxidized/reduced) through doping. A significant amount of research has been undertaken to enhance the thermal conductivity of these polymers by modifying the arrangement of the chains within the network or altering their chemical structures. When polymers are converted into nanofibers, their thermal conductivity increases significantly, reaching approximately 104 W/m·K. This is because the polymer chains align into an “ideal” single crystalline form once they are stretched. For example, polyethylene (PE) nanofibers with diameters between 50 and 500 nm and lengths of several millimeters have shown an increase in thermal conductivity from 0.5 W/m·K to 104 W/m·K [19]. Polythiophene nanofibers produced via templated electropolymerization have also been found to exhibit up to 20 times better thermal conductivity, particularly those with a diameter of 50 nm [20].

Selecting specific ligands and metal ions can have a significant impact on the structure, stability, and other properties of the metal–organic assemblies formed [21,22,23,24,25]. Organometallic polymers are an important type of material that emerged in the 20th century. They are becoming more popular because they are strong, flexible, and relatively inexpensive to produce [26]. The most remarkable attribute about these organometallic systems is that they combine organic and inorganic parts in a manner that no other system accomplishes. Organometallic polymers are being used increasingly in a wide range of fields, from electronics and optoelectronics [27,28,29,30] to sensory materials [31,32,33,34]. Polyacid polymers are among the most popular types of ligands used to make organometallic polymers. The chemical structure of polyacids, including the types of acidic groups, the arrangement of the polymer chain, and the distance between the main chain and the acidic sites, significantly influences their functionality and potential applications. Polymers in this category typically exhibit hydrophilic properties, can conduct protons, easily form complexes with metal ions, and demonstrate biocompatibility.

Our research concentrates on developing new organometallic polymers with enhanced crystallinity and thermal conductivity. A key aspect of this work is employing two important synthetic methods to synthesize novel organometallic frameworks. These methods include solution-based synthesis and mechanochemical reactions, with a particular emphasis on the principles of green chemistry. These new complexes are anticipated to exhibit interesting chemical and physical properties due to their incorporation of metal ions into the polymer structure. Such properties include high thermal conductivity, elasticity, and tunable porosity, which may lead to new applications.

## 2. Materials and Methods

### 2.1. Materials

All the starting materials used in this study were purchased from Sigma-Aldrich (St. Louis, MO, USA) and Alfa Aesar by Thermo Fisher Scientific (Waltham, Germany) and did not require further purification. **PBAAA** was synthesized according to the literature [35]. Heating 4-hydroxymandelic acid at 160 °C for 15 h induces Friedel–Crafts polycondensation, leading to a new polymer, **PBAAA**, rather than the polyester formation typical for α-hydroxy acids. In the resulting **PBAAA**, phenol units are linked by carboxymethylene bridges. Additionally, partial lactonisation occurs through the reaction of the phenolic OH with carboxyl groups, forming benzofuranones and contributing to the structure of **PBAAA**.

#### 2.1.1. Synthesis of Polyaryacetic Acid **PAAA**

To synthesize the polyaryacetic acid ligand **PBAAA**, 1 g of polymer **PBAAA** was dissolved in a solvent mixture of 40 mL water and 40 mL ethanol. Next, a separate solution was prepared by diluting 25 mL of 25% ammonia solution (NH_4_OH) in 25 mL of water. Upon combining the two solutions, a colorimetric shift from purple to blue was observed during stirring at the ambient temperature. After 1 h, the solvent was evaporated under reduced pressure, yielding a dark brown powder identified as **PAAA**.

#### 2.1.2. General Procedure for Traditional In-Solution Synthesis of Organometallic Polymers

1 g of the polymer ligand was dissolved in 30 mL of water and 30 mL of ethanol. An aqueous solution of metal acetate (Zn(CH_3_CO_2_)_2_∙2H_2_O, Cu(CH_3_CO_2_)_2_∙H_2_O and Co(CH_3_CO_2_)_2_∙4H_2_O) (2, 1, or 0.5 equivalents, 20 mL) was then introduced to the mixture while stirring at room temperature. The addition of the metal acetate resulted in the formation of a precipitate. The reaction mixture was stirred for an additional hour at room temperature. Afterwards, the precipitate was carefully filtered and subsequently washed with water and ethanol.

#### 2.1.3. General Procedure for Mechanochemistry Synthesis of Organometallic Polymers

In a 35 mL zirconia jar, a single 20 mm diameter ZrO_2_ ball was introduced along with 1 g of ligand polymer, a metal salt (in a 1:1 mass ratio), and 8 g of NaCl. The jar was subsequently placed in a milling machine, where it underwent mechanical milling for durations of 1, 2, or 4 h at a frequency of 30 Hz. Following milling, a mixture of water and ethanol was added to the resultant crude mixture. The precipitate was then filtered and thoroughly washed with both water and ethanol to ensure purity.

### 2.2. Methods

The FTIR spectra were measured by a JASCO FTIR spectrometer model 6100 (Tokyo, Japan) in the 5000–350 cm^−1^ spectral range using KBr (IR grade) pellets technique.

UV-Vis absorption spectra were carried out on a Cecil Super Aquarius spectrophotometer (UV-1100) using quartz cuvettes (1 cm).

X-ray diffraction patterns were obtained at ambient temperature utilizing a Rigaku SmartLab multipurpose diffractometer operating with Cu Kα1 radiation (λ = 1.54056 Å) and equipped with a 9 kW rotating anode. The experimental data acquisition was facilitated by SmartLab Guidance software (version 2.1.0.0). Measurements were systematically carried out over an angular range of 5° to 90° with a step increment of 0.01°.

Thermogravimetry measurements were performed in air with 12% O_2_ in N_2_, using TA Instruments SDT Q600 equipment, with a temperature range of 30 to 1000 °C and a heating rate of 10 °C/min.

The thermal conductivity was measured using a Hot Disk TPS 2500 S (Hot Disk AB, Kagaku, Sweden) apparatus with a 5464F1 sensor, employing the transient plane source (TPS).

XPS spectrometer from SPECS with an Al/Mg dual-anode X-ray source, PHOIBOS 150 2D CCD hemispherical energy analyzer, a multichanneltron detector and the pressure in the vacuum chamber was maintained at 1 × 10^−9^ Torr in order to perform XPS analysis. The X-ray source (1486.6 eV) was operated at a power of 200 W while the XPS survey spectra were recorded at 30 eV pass energy and 0.5 eV/step. The high-resolution Cu2p spectra were recorded for each sample by accumulating 10 scans at 30 eV pass energy and 0.1 eV/step. Data analysis and deconvolutions were performed using Casa software (6.7) with a Gaussian–Lorentzian product function (GL30) after the nonlinear Shirley background subtraction from the obtained data. It is good to note that the spectra obtained before being analyzed were calibrated in order to correct for peak shifts due to any apparent charging, relative to C1 s peak set to 285 eV.

## 3. Results and Discussion

### 3.1. Synthesis and Spectroscopic Characterization of Polymeric Ligands

#### 3.1.1. Synthesis of Polymeric Ligands

**Scheme 1:**
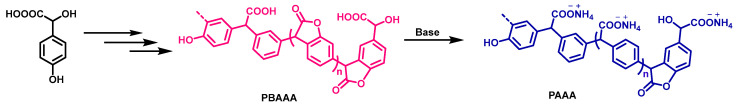
Synthesis of **PBAAA** and **PAAA** polymers.

For this study, we synthesized **PBAAA** via a polycondensation process, heating 4-hydroxymandelic acid without the use of solvents or catalysts. Unlike other α-hydroxy acids, which usually produce polyesters, 4-hydroxymandelic acid underwent a Friedel-Crafts alkylation, forming C-C bonds between phenolic units. This method yielded polymers with both carboxylic and phenolic groups. There was also partial lactonisation, occurring when the phenolic hydroxyl groups and carboxylic groups interacted, forming benzofuranones. As a result, this approach produced poly(benzofurane-co-arylacetic acid) structures of polymer ligand **PBAAA** (Scheme 1).

#### 3.1.2. FTIR Spectroscopy of Polymeric Ligands

**Figure 1:**
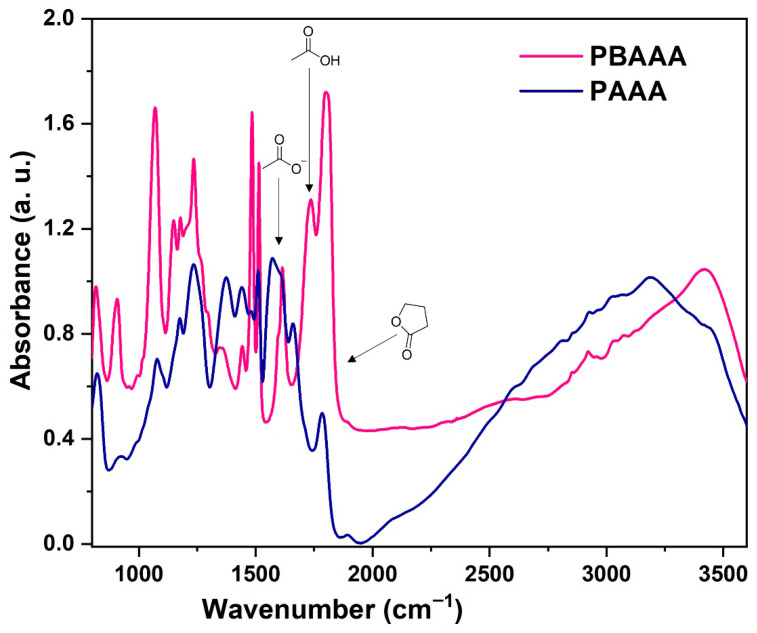
FTIR spectra of **PBAAA** and **PAAA** polymeric ligands.

The structure of the polymer **PBAAA** ligand is highly dependent on pH, and bases can easily open the lactone ring. Treating **PBAAA** with ammonia solution primarily facilitates the opening of these lactone rings, resulting in the formation of the ammonia salt of polyaromatic acetic acid, known as **PAAA** (Scheme 1). Figure 1 presents the FTIR spectra for both **PBAAA** and **PAAA** polymer ligands. The different positions and intensities of the absorption bands in these spectra indicate that PBAAA and **PAAA** have significantly different structures. The stretching vibrations of the -OH group, which are present in the carboxyl group of the polymer as well as those from adsorbed water on the polymer chain, provide distinct absorption bands in the region of 2500–3500 cm^−1^. In the case of **PAAA**, this adsorption band is broader than that seen in the **PBAAA** spectrum. This broadening occurs because the number of carboxylic groups increases as the lactone ring opens and transforms into -COOH or -COO^−^ groups. At this wavenumber, the **PAAA** polymer also exhibits absorption bands associated with the ammonium groups (-NH_3_), which act as counter-ions to the carboxylic acid. The FTIR spectrum of **PBAAA** shows strong bands related to lactone rings at 1800 cm^−1^ and protonated carboxylic groups at 1734 cm^−1^. In contrast, the FTIR spectrum of **PAAA** reveals significant absorption bands corresponding to deprotonated carboxylic acid groups at around 1662 cm^−1^ and 1594 cm^−1^. The carbonyl absorption related to the lactone in the **PAAA** spectrum appears at a lower wavenumber with decreased intensity, indicating an overlap with the absorption band characteristic of the -COOH group. Ammonia promotes the cleavage of the lactone ring, which is evidenced by the decrease in the absorption band. The adsorption bands observed in both FTIR spectra, at 1615 and 1517 cm^−1^ for **PBAAA** and 1564 and 1510 cm^−1^ for **PAAA**, are assigned to aromatic C=C stretching vibrations, reflecting the aromatic structure of the polymers. For **PBAAA**, additional important adsorption bands are present at 1483 cm^−1^ and 1442 cm^−1^ and are attributed to C–H bending from the aromatic ring. In **PAAA**, these bands shift to lower wavenumbers at 1429 cm^−1^ and 1372 cm^−1^, describing both aromatic C-H bending and the -CH_2_ group. In both FTIR spectra, a band around 1236 cm^−1^ corresponds to C–O stretching vibrations of the carboxylic acid. The sharply defined and intense adsorption band observed in the **PBAAA** FTIR spectrum at 1068 cm^−1^ is assigned to the C–O stretch of the benzofuran ether group. This same band appears in the FTIR spectrum of **PAAA** but with much less intensity. Furthermore, bands at 912 and 818 cm^−1^ in the **PBAAA** FTIR spectrum correspond to out-of-plane C–H bending vibrations of aromatic rings. Although these bands are also present in the **PAAA** spectrum, they are less intense, the band at 912 cm^−1^ is barely detectable, and the one at 818 cm^−1^ is also weaker. It is worth mentioning that the benzofuran introduces distinctive ether-like C–O stretches and ring-breathing vibrations, distinguishing **PBAAA** from the simpler aromatic polymer **PAAA**.

#### 3.1.3. Powder XRD of Polymeric Ligands

**Figure 2:**
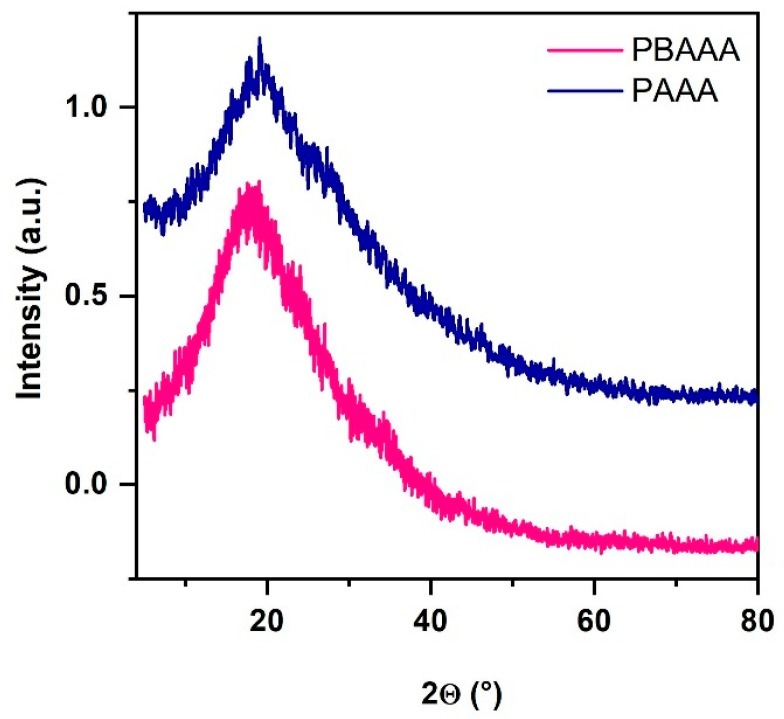
Powder XRD pattern of **PBAAA** and **PAAA**.

Figure 2 shows the powder XRD analysis of the ligand polymers **PBAAA** and **PAAA**. The diffractograms provide important data about the degree of order within the polymer matrix. Crystalline materials typically produce sharp, well-defined peaks in XRD due to their long-range atomic order. In contrast, broad peaks are associated with lower crystallinity or the presence of nanocrystalline domains in the analyzed material. The XRD pattern attributed to the **PBAAA** ligand shows that polymer **PAAA** has a peak around 20° 2θ. This diffraction pattern indicates a semi-crystalline structure with limited long-range order, suggesting the presence of amorphous regions and small crystallite sizes, which is characteristic of polymers. Similarly, ligand polymer **PAAA** exhibits a prominent peak near 20° 2θ, indicating a more crystalline structure, which implies a more effective molecular arrangement. The sharper peak observed for **PAAA** supports the idea of well-defined crystalline domains. No clear peaks are observed in the **PAAA** and **PBAAA** polymers. Therefore, no crystalline size can be determined, but their degrees of crystallinity can be compared by deconvolution of the XRD graphs, as shown in Figures S1 and S2. The crystallinities of **PBAAA** and **PAAA** were determined to be 32% and 35.4%, respectively.

#### 3.1.4. UV-Vis Spectroscopy of Polymeric Ligands

**Figure 3:**
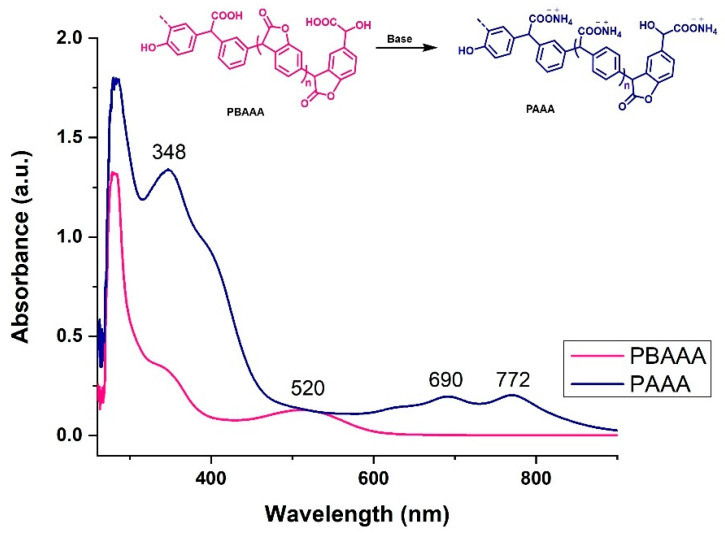
UV-Vis spectra of **PBAAA** and **PAAA** measured in DMSO.

We also investigated the structural changes in the polymers **PBAAA** and **PAAA** by UV-Vis analysis. The polymer **PBAAA** displays two bands at 284 nm, a falling shoulder at 355 nm, and another peak at 520 nm. This assignment is associated with the π-π* transitions of the aromatic units in the repeat unit and with the internal charge transfer (ICT) processes occurring between groups of the polymer core and the electron acceptor groups (Figure 3). Upon the addition of NH_4_OH to a solution, the absorption bands at 284 and 355 nm are enhanced, and two new absorption bands are present in the region of far-red and infrared. These alterations are attributed to a higher ordered structure and better conjugation along the polymer backbone. In contrast to **PBAAA**, the UV-vis spectra of **PAAA** show the characteristic absence of the peak at 520 nm. The enhanced electron-withdrawing effect of the carboxylic group in **PAAA**, in comparison to the lactone group in **PBAAA**, is considered responsible for this effect. As a result, ICT within the **PAAA** polymer is enhanced, resulting in a red shift of the absorption maximum compared to **PBAAA**. UV-Vis spectroscopy was also used to monitor the synthesis of polymer ligands **PAAA** from **PBAAA** (Figure S3).

#### 3.1.5. TGA of Polymeric Ligands

**Figure 4:**
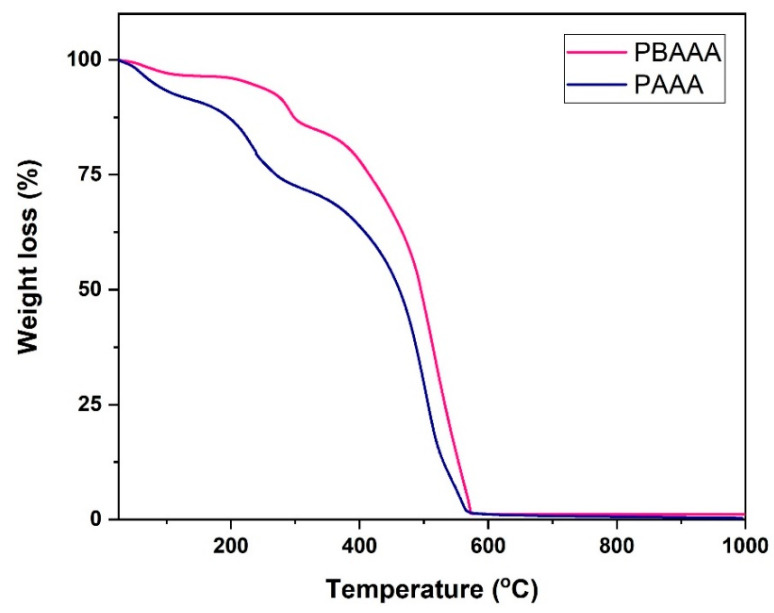
Thermogravimetric curves for the **PBAAA** and **PAAA**, each performed in air at a heating rate of 10 °C/1 min between 30 °C and 1000 °C.

The thermal stability of **PBAAA** and **PAAA** was investigated by TGA (Figure 4). The ligands decompose in three and four steps, respectively. In the first two steps (25–150 °C), the loss of both coordination and absorption water molecules takes place for both compounds, accounting for a 4.5% and 6.9% mass loss for ligand **PBAAA** and **PAAA**, respectively. Further, one decomposition step is observed for **PBAAA**, and two decomposition steps are observed for **PAAA**, which, according to the mass losses, 10.2% and 19.2% correspond to the breaking of carboxylic groups and lactone rings. The last decomposition steps (85.3% for **PBAAA** and 73.9% for **PAAA**) correspond to the breaking and burning of the aromatic bonds of the polymer. The final decomposition temperature for both compounds, **PBAAA** and **PAAA**, is 590 °C. Generally, the conclusion is that **PBAAA** exhibits greater heat resistance stability due to its lactone moiety, in contrast to the carboxylic ammonium salt from **PAAA**, which shows reduced stability.

### 3.2. Synthesis and Characterization of Organometallic Polymers

#### 3.2.1. Synthesis of Organometallic Polymers

The polymer PBAAA served as a ligand for coordinating with Zn(CH_3_CO_2_)_2_·2H_2_O, Cu(CH_3_CO_2_)_2_·H_2_O, and Co(CH_3_CO_2_)_2_·4H_2_O, resulting in slightly basic aqueous solutions that facilitated the in situ ring opening of predominantly lactone rings, thereby generating carboxyl groups suitable for coordination. For comparative purposes, PAAA, synthesized via reaction with an ammonium solution, was also employed in specific complexation experiments. We systematically investigated the influence of various operating parameters on the composition of the resultant polymer–metal complexes. This included examining the ligand-to-metal salt ratio, the impact of different synthetic methodologies (solution-based versus solid-state synthesis), and the effect of reaction time during ball-milling synthesis. Structural characterization of the synthesized metal complexes was performed through FTIR, X-ray powder diffraction, and thermogravimetric analysis (TGA). Additionally, we correlated these structural findings with the thermal conductivity properties of the novel materials, contrasting them with those of the uncoordinated polymeric ligands.

Organometallic polymers of Co^2+^, Cu^2+^, and Zn^2+^ were synthesized utilizing both solution and solid-state approaches. To elucidate the coordination behavior of polymeric ligands with metal salts, various synthesis parameters were optimized. In the solution-based method, the ligand-to-metal salt ratio was carefully adjusted for PBAAA and copper acetate, yielding three distinct complexes. It was postulated that each monomeric unit of **PBAAA** harbors a free carboxylic or lactone group capable of coordinating with metal ions. The synthesis involved the addition of an aqueous metal acetate solution to a **PBAAA** ligand solution in a water-ethanol mixture or to an aqueous solution of **PAAA** at ambient temperature, resulting in the formation of a precipitate. This precipitate was then filtered, washed with water and ethanol, and dried.

**Scheme 2:**
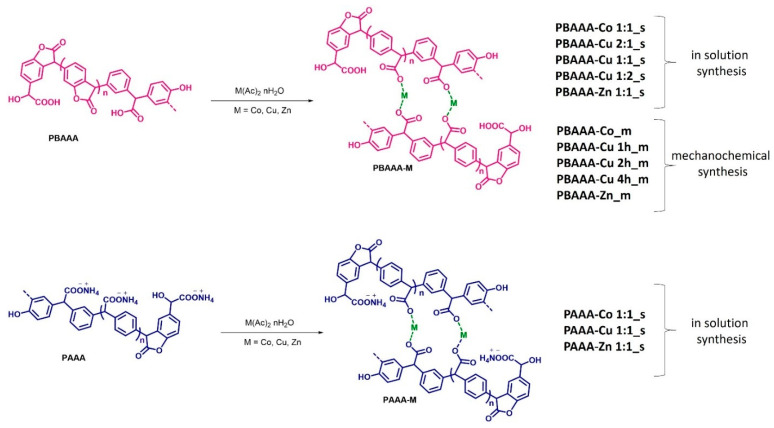
The synthesis of Co, Cu and Zn metal complexes of **PBAAA** and **PAAA**.

For the solid-state method, the effect of reaction time on the complexation process was investigated. It was found that mechanical treatment of the polymers could lead to bond cleavage, hence, three different milling durations were examined for **PBAAA** and Cu(CH_3_CO_2_)_2_: 1 h, 2 h, and 4 h [36,37]. This study aimed to investigate the effect of varying milling time on the properties of the resulting polymers (Scheme 2).

#### 3.2.2. FTIR Spectroscopy of Organometallic Polymers

All organometallic polymers were analyzed using FTIR spectroscopy to investigate the influence of metal ions on the polymer structure. Figures S4–S8 present the FTIR spectra of all the organometallic polymers synthesized in this study. The information obtained from these spectra indicates that the formation of a complex with a metal ion leads to noticeable changes in the FTIR spectral adsorption bands. The most significant changes are observed in the FTIR spectra of the **PBAAA** polymer, which is used as a ligand. A clear shift and decrease in the intensity of the adsorption bands are evident, suggesting that the metal ion used as a complexing agent opens the lactone rings. Specifically, the band at 1800 cm^−1^ in the FTIR spectrum of **PBAAA**, attributed to the C=O bond from the lactone ring, shifts to 1790 cm^−1^ and shows reduced intensity in the presence of metal complexes. The specific band associated with carboxyl groups at 1735 cm^−1^ remains visible, albeit with very low intensity, only in the case of **PBAAA-Co 1h_m**. In contrast, this band disappears completely for the other complexes due to the transformation of carboxyl groups (-COOH) into carboxylate (COO^−^) anions. The band assigned to the carboxylate groups becomes extremely intense and broad in all polymeric complexes of **PBAAA** at 1608 cm^−1^, indicating the successful complexation of all metals by the polymeric ligands. The adsorption band at 1068 cm^−1^ is assigned to the C–O stretch of the benzofuran ether group in the FTIR spectrum of **PBAAA**, which significantly decreases across all metal complexes (Figures S4–S7).

In the case of PAAA used as a ligand for complexation, the changes observed in the FTIR spectra are not as pronounced as those seen with PBAAA (see Figure S8). The most significant change in the FTIR spectra is the shift of a very broad band, initially centered at 3188 cm^−1^, to a higher wavenumber of 3400 cm^−1^. This shift is attributed to the replacement of ammonium ions and -OH groups by the metal ion.

Another noticeable change occurs in the absorption band specific to the -COOH group at 1735 cm^−1^, which is less intense in the metal complexes compared to the pure PAAA. Consequently, the absorption bands corresponding to the deprotonated carboxylic acid groups, initially present at around 1662 cm^−1^ and 1594 cm^−1^ in the PAAA FTIR spectra, merge into a single band that appears at 1590 cm^−1^ in the case of complexing the polymer with the metal ions.

#### 3.2.3. Powder XRD of Organometallic Polymers

**Figure 5:**
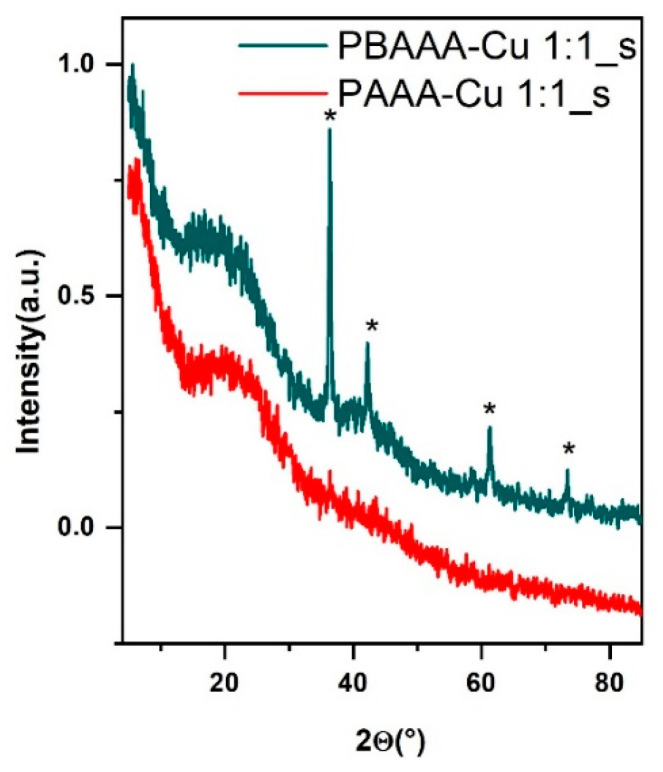
XRD pattern of Cu complex using **PBAAA** and **PAAA** as ligands when the reaction was performed in solution; * Cu_2_O—DB card number 01-071-3645.

**Figure 6:**
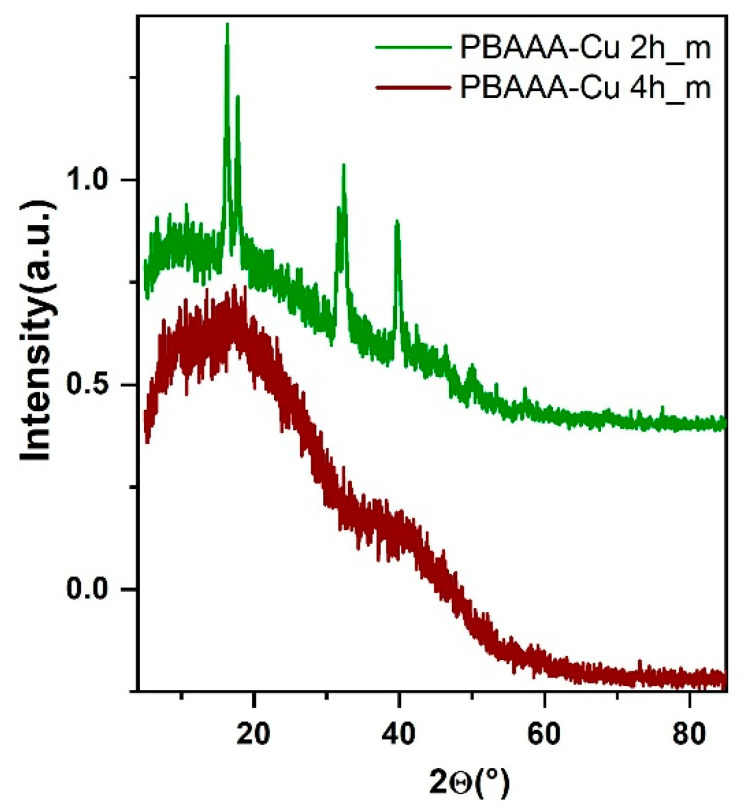
XRD pattern of Cu complex using **PBAAA** as ligand after 2 h and 4 h of mechanosynthesis.

The X-ray diffraction (XRD) analysis reveals the generation of amorphous phases across all metal complexes of the polymers, characterized by varying degrees of crystallinity. When polymer **PBAAA** reacts with copper acetate, copper(I) oxide is formed as a byproduct, irrespective of the metal–ligand stoichiometry (illustrated in Figure 5). Notably, the formation of Cu_2_O as a byproduct is absent when polymer **PAAA** is utilized as the ligand (compound **PAAA-Cu 1:1_s**) or in solid-state synthesis (compounds **PAAA-Cu 2h_m**), as shown in Figure 6.

The mechanosynthesis method yielded distinct products with diverse crystallinity profiles compared to those obtained through solution-based synthesis. Our initial focus was on optimizing reaction times for mechanosynthesis. X-ray diffraction (XRD) patterns for the Cu metal complexes processed for 1 h (**PBAAA-Cu 1h_m**) and two hours (**PBAAA-Cu 2h_m**) reveal well-defined crystalline peaks that coexist with amorphous peaks, indicating a hybrid structure with both crystalline and amorphous characteristics (see Figure 6). In contrast, the diffractogram for the 4 h processed sample (PBAAA-Cu 4h_m) exhibits a completely amorphous phase (Figure 6). This finding supports the inference that extended reaction times in mechanochemical processing lead to the degradation of the polymeric chains within the ligand.

#### 3.2.4. Thermogravimetric Analysis of Organometallic Polymers

In Figure S7, the derivatograms and thermogravimetric curves for both the polymers and all related polymer–metal complexes are presented. The thermal properties of the synthesized metal complexes have been evaluated using thermogravimetric curves, the significant data being summarized in Table 1, Table 2 and Table 3. Complexes obtained by reacting PBAAA and a metal salt in solution display two or three distinct decomposition stages. The initial mass loss, occurring between approximately 150–200 °C, is due to the elimination of inter- and intra-molecular water molecules absorbed within the complexes. The following decomposition phase, associated with the breakdown of carboxylic and lactone groups, occurs up to around 300–320 °C in **PBAAA-Cu** complexes and extends up to 420 °C in complex **PBAAA-Zn**. The final decomposition temperatures for **PBAAA-Cu** complexes are situated between 478–485 °C, and at 520 °C and 893 °C for **PBAAA-Co 1:1_s** and **PAAA-Co 1:1_s**, respectively. The residual mass after decomposition ranges between 11% and 19%. In line with findings from other metal complexes, the remaining residue indicates the formation of metal oxide structures, and further analyses will be performed to clarify their structural and functional properties [38,39,40,41].

Notably, for **PBAAA-Cu** organometallic compounds, a gradual increase in residual mass is observed from **PBAAA-Cu 2:1_s** to **PBAAA-Cu 1:1_s**, while **PBAAA-Cu 1:2_s** shows no further increase. This suggests that using excess metal salt does not significantly change the chemical composition of the complex, implying that a 1:1 coordination equilibrium between the carboxyl groups and the metal center has been established.

The as-synthesized compounds involving Co^2+^, Cu^2+^, and Zn^2+^ with PAAA exhibit two to three distinct thermal decomposition phases. The final decomposition temperatures for the complexes **PAAA-Co 1:1_s**, **PAAA-Cu 1:1_s**, and **PAAA-Zn 1:1_s** are 455 °C, 495 °C, and 545 °C, respectively. The residual mass percentage after decomposition ranges from 17% to 26%, with no clear correlation to the ionic radii of the metal cations. The derivatograms of the cobalt complexes **PBAAA-Co 1:1_s** and **PAAA-Co 1:1_s** display an additional stage in the decomposition process, resulting in approximately 1% mass loss at 900 °C. This observation aligns with findings in compounds synthesized via mechanochemistry.

The derivatograms for compounds synthesized by mechanochemistry (see Figure S9) show that there are four to five separate phases in the breakdown process, with the final temperatures of decomposition ranging from 860 to 992 °C. These temperatures are significantly higher than those observed when the same chemicals were prepared in solution. The results indicate that mechanochemical synthesis methods yield distinct coordination complexes, which differ from those formed in solution-phase processes.

#### 3.2.5. Thermal Conductivity of Organometallic Polymers

The thermal conductivity (κ) of polymeric complexes can be influenced by various factors, including the structure of the monomer, the crystalline arrangement of the chains, as well as the chain conformations, morphologies, and topologies. This study investigates some of these factors. We report κ measurements taken at room temperature, 50 °C, and 100 °C on a series of polymer complexes. Utilizing the hot disk technique allows for rapid and high-accuracy measurements of the thermal transport properties of polymers.

The aim of this study was first to examine the dependency of κ on ionic radius, thereby verifying the previously predicted theoretical correlation, and second to analyze the impact of the synthesis method, the degree of crystallinity, and the ligand-to-metal ratio on κ.

**Figure 7:**
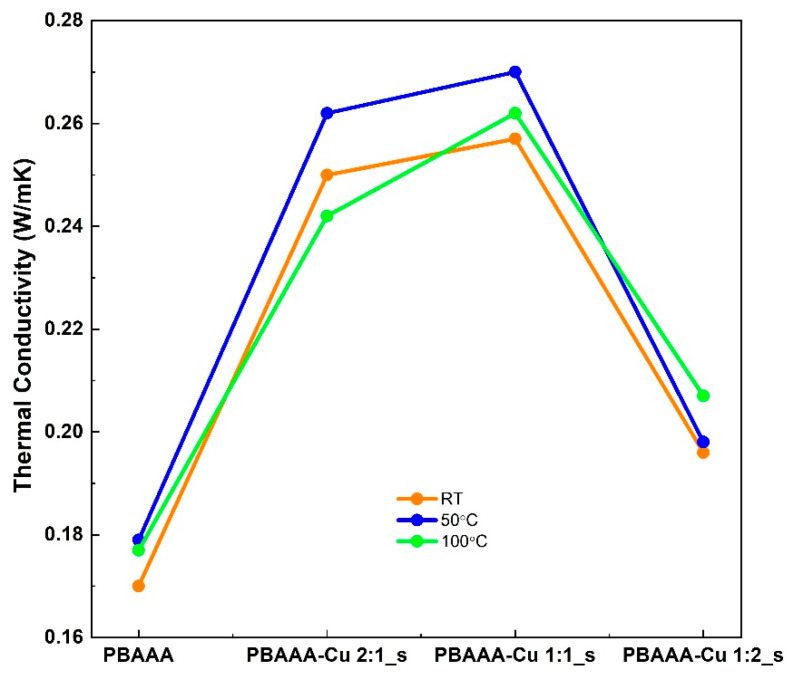
Thermal conductivity as a function of the ligand **PBAAA** and copper salt ratio.

Figure 7 presents the thermal conductivities of the synthesized polymer complexes with varying ligand-to-metal ratios and compares them with the properties of the free ligand (PBAAA). The presence of copper coordinated to the polymer significantly enhances κ, doubling its properties, which is closely related to the amount of copper used in synthesis. The results indicate that thermal conductivity increases with the metal ion content, ranging from 0.17 W/mK to 0.25 W/mK for a ligand-to-metal ratio of 1:1. However, with a further increase in the metal ion ratio, the κ decreases to 0.19 W/mK. This observed behavior in κ is also consistent across measurements taken at higher temperatures of 50 °C and 100 °C.

**Figure 8:**
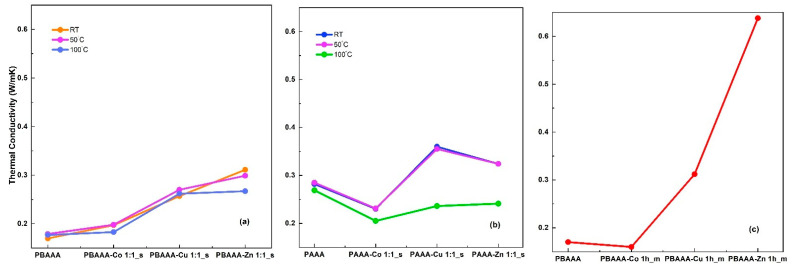
(**a**) κ variation in PBAAA–metal complexes when reactions were carried out in solution; (**b**) κ variation in PAAA–metal complexes; (**c**) κ variation in PBAAA–metal complexes when reactions were carried out using mechanochemistry.

Achieving tunable κ can be done by changing the metal ions or the synthesis method. Mechanochemistry is an energy-efficient technique that involves mechanical force to promote chemical transformations [42,43]. The most important advantages of using it are solvent-free conditions, energy savings, and increased reactivity [44,45]. During the milling process, the milling balls provide a high-impact energy, which can polymerise activated monomers directly without using an external initiator. Figure 8 shows that after milling for 1 h and 2 h, respectively, the obtained coordination polymer has an increased κ (0.32 W/mK), almost double that of the initial compound (0.16 W/mK), which is a confirmation of the fact that the obtained polymer has a more ordered structure of the chains. Then, if the milling process continues, after 4 h, the thermal conductivity decreases to a value that is even slightly lower than the initial one (0.14 W/mK). This indicates that the polymer undergoes a decomposition or degradation process when exposed to prolonged mechanical friction forces. Therefore, our data demonstrate that there is an optimal exposure time in the milling process during which the complex forms without destroying the polymer chains. Beyond this point, if the process continues, the polymer begins to decompose or degrade.

Another influential factor examined in this study was the effect of ionic radius on the conductive network. It is known that the ionic radius is determined by interatomic interactions driven by Van der Waals and Coulomb forces. Thus, a smaller ionic radius causes a shorter distance of interaction between a counterion and the ionized group of polymers, a correlation that is also expected to be observed in the thermal conductivity measurements. Figure 8 shows the thermal conductivity measured for the samples containing different types of ions: Co^2+^, Cu^2+^, and Zn^2+^. We observe an increasing trend in the obtained data, consistent with previous findings on polyelectrolytes with different counterions using molecular dynamics simulations [42], which indicate a strong negative relationship between κ and ionic radii.

Figure 8 illustrates how κ varies with different types of ions. It shows that κ consistently increases as the ionic radius of the metal ion decreases. Previous studies have shown that this trend is associated with a decrease in ionic radius. It is possible that the coordination numbers of the ions in the resulting molecule may also influence this relationship. Consequently, the data allows us to assign precise values to the ionic radii and coordination numbers (CNs) of the ions involved, as shown in Table 4.

The coordination number refers to the number of chemical bonds formed between a metal ion and its ligands. Each CN is associated with one or more geometric arrangements. Therefore, understanding the CN helps determine whether the resulting compounds are ionic or molecular. For instance, a CN of 5 indicates that the ion interacts with five neighboring atoms or ligands. The possible geometries for this CN include square pyramidal and trigonal bipyramidal shapes. Coordination number 6 is the most common among all compounds, with the preferred geometry typically being octahedral (either regular or distorted) in addition to the trigonal prism geometry.

By applying the same analytical approach to Figure 8b, we can conclude that using the ligand **PAAA** alters the radius of the metal ions, which consequently affects the coordination geometry and the number of ligands due to the different nature of the resulting compounds.

Figure 8c shows how κ changes depending on the type of metal ion in the polymer complexes synthesized through mechanochemistry. A significant increase in thermal conductivity is observed compared to the values obtained for polymer complexes prepared in solution (see Figure 8a,b). Additionally, similar to the observations in Figure 8a, this behavior correlates with the variation in the radius of the metal ions in the samples analyzed. Therefore, using the same ligand likely results in the formation of the same chemical compounds via both mechanochemistry and solution synthesis. The increased conductivity values may be attributed to the preservation of structural order over greater distances when employing the mechanochemical synthesis method, as indicated by the powder XRD data.

#### 3.2.6. X-Ray Photoelectron Spectroscopy of Organometallic Polymers

To gain a deeper understanding of the changes in the chemical characteristics of the analyzed samples, we conducted a detailed examination of PBAAA-Cu 1:1_s and PAAA-Cu 1:1_s complexes using XPS.

**Figure 9:**
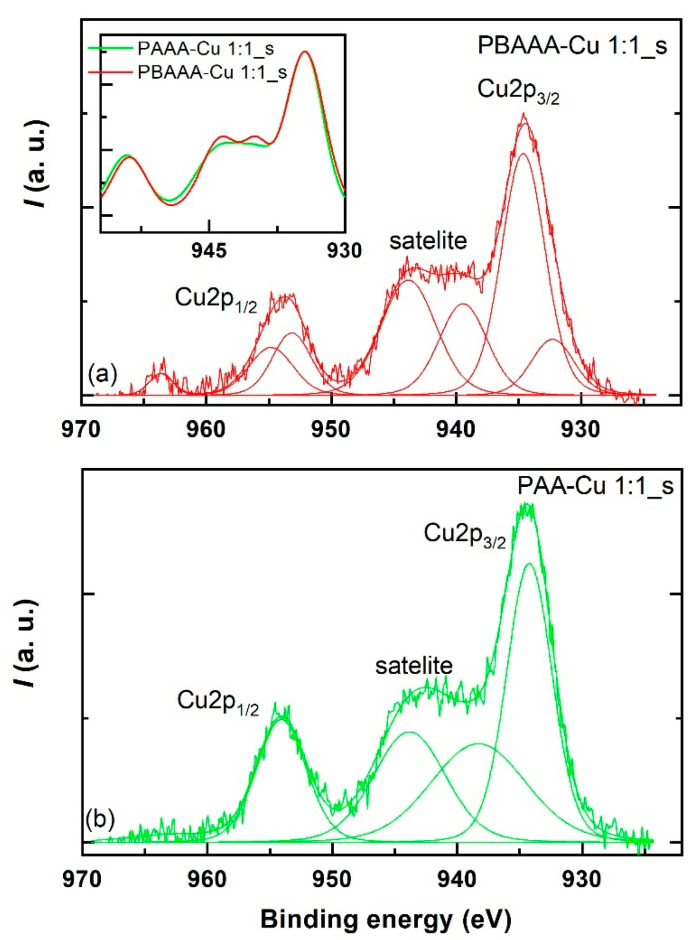
XPS spectra of complexes (**a**) **PAAA-Cu 1:1_s** and (**b**) **PBAAA-Cu 1:1_s**.

The Cu 2p spectra for the PAAA-Cu 1:1_s and PBAAA-Cu 1:1_s complexes are shown in Figure 9a,b. The spectra display the characteristic spin–orbit split doublets corresponding to the Cu 2p1/2 and Cu 2p3/2 lines, along with the typical satellite features associated with Cu^2+^ compounds [47].

The Cu2p spectra for the **PAAA-Cu 1:1_s** and **PBAAA-Cu 1:1_s** organometallic polymers are displayed in Figure 9a,b. The spectra feature spin–orbit doublets corresponding to the Cu 2p1/2 and Cu 2p3/2 lines, along with the satellite structure typical of Cu^2+^ compounds.

In Figure 9a, the main photoemission line for the **PBAAA-Cu 1:1_s** organometallic polymer shows a slight asymmetry in the low binding energy region. This asymmetry can be attributed to the inclusion of an additional peak corresponding to a Cu^+^ chemical state. For this sample, the signal attributed to Cu^2+^ comprises 82% of the total integrated intensity. Conversely, the **PAAA-Cu 1:1_s** compound reveals deconvolution components that can be attributed solely to Cu^2+^ species. The relative atomic percentages of Cu^+^ and Cu^2+^ are provided in Table 5, indicating a value of 4.8 for the **PBAAA-Cu 1:1_s** sample.

The inset of Figure 9a presents a direct comparison of the normalized Cu2p XPS spectra for the samples under study. The graphs show a significant difference in the satellite region, which is associated with the electronic structure of the complexes [48]. This satellite region is typically present in all materials with a ground-state configuration and is directly associated with the multiple splitting in the 2p 3d final state. It has been well established that Cu_2_O has an open-shell (3d) configuration and is a semiconductor with a band gap of approximately 1.4 eV [49]. This characteristic arises due to electron correlation. However, it is classified as a charge transfer type since the d-d Coulomb interaction surpasses the charge transfer energy [50].

As illustrated in the graph, in the case of **PBAAA-Cu 1:1_s**, the Cu2p spectrum satellites have higher intensity and are more distinctly separated from the main line compared to the **PAAA-Cu 1:1_s** sample. Furthermore, the satellite region in **PBAAA-Cu 1:1_s** is split, unlike in **PAAA-Cu 1:1_s**, where it exhibits a broad shoulder shape. This observation can be explained by the previously reported theoretical study, which is based on the fact that satellites arise solely from the excitation of unpaired electrons [48]. This allows the attribution of the split satellite in **PBAAA-Cu 1:1_s** to excitation of an unpaired electron from the ligand to high-lying orbitals localized on copper. Conversely, the satellite with a continuous, unsplit line in **PAAA-Cu 1:1_s** may be attributed to excitation of the unpaired electron to high-lying orbitals delocalized on ligands.

These findings align well with the results on thermal conductivity, which are also closely linked to the density of unpaired electrons and the strength of bonding between copper atoms within the structure, among other factors. The results indicate that **PAAA-Cu 1:1_s** has a lower thermal conductivity compared to **PBAAA-Cu 1:1_s**, which contains the ligand with the highest number of delocalized electrons. As a result, it is more likely that unpaired electrons in specific copper orbitals will get excited during photoionization. This effect contributes to the observed splitting in the satellite region of the XPS spectrum.

## 4. Conclusions

In the work, the successful preparation and characterization of the novel organometallic polymeric complexes based on lactone-containing poly(benzofuran-co-arylacetic acid) (**PBAAA**) and its ammonium salt (**PAAA**) with Co^2+^, Cu^2+^ and Zn^2+^ ions have been demonstrated. Both conventional solution-based and mechanochemical ball milling routes were used and systematically compared. Mechanochemical synthesis has proven to be a green, robust, and effective alternative to traditional solution-based procedures, allowing solution-free reactions and facilitating energy savings while overcoming solubility issues. This approach enabled the controlled variation in polymer properties by adjusting the milling time. XRD data revealed that mechanosynthesis resulted in a higher structural order and a higher degree of crystallinity compared to solution synthesis. In addition, XRD measurements showed that the optimal mechanochemical milling duration was 1–2 h, which allowed for adjustments between better coordination and chain ordering, as well as polymer degradation, at longer milling times.

This study demonstrates that the thermal conductivity increases as the ionic radius decreases for the studied complexes; however, in the case of the organometallic polymer **PAAA-Cu 1:1_s**, where Cu ions were used in solution with **PAAA** as a ligand, a lower thermal conductivity value is observed compared to the use of ligand **PBAAA**, which has the highest number of delocalized electrons. This finding supports the XPS results, which show a splitting of the satellite region in this specific sample due to the excitation of the unpaired electron. Furthermore, the mechanical synthesis method yields more efficient and sustainable outcomes, overcoming solubility limitations and facilitating the incorporation of diverse components. The thermal conductivity measurement indicates that the resulting coordination polymer exhibits an increased thermal conductivity (0.32 W/mK), nearly double that of the initial compound (0.16 W/mK), after milling for 1 h and 2 h, respectively. In conclusion, the mechanochemical synthesis method is a promising green chemistry approach compared to traditional solution synthesis, as it eliminates the need for solvents, consumes less energy, and enables easy modification of material properties by adjusting the milling parameters. Following a comprehensive analysis of the thermal properties of all three metal–polymer complexes, including TGA and thermal conductivity measurements, it was observed that the Zn polymer complexes demonstrated a relatively good thermal stability and an excellent thermal conductivity. Co-polymer complexes provide superior thermal stability, but not significantly high thermal conductivity. Conversely, the Cu polymer complexes exhibited low levels of thermal stability and conductivity. These insights guide the selection of metal ions for tailoring the properties of organometallic polymers to meet specific thermal applications.

In summary, this work demonstrates that mechanochemical synthesis is an attractive green chemistry tool for preparing organometallic polymers with adjustable and enhanced thermal properties. The study provides valuable insights into the interplay between polymer structure, metal coordination, and thermal transport, thereby establishing a new platform for developing advanced functional materials for electronic, sensing, and thermal management systems.

## Figures and Tables

**Table 1:** TGA data for metal complexes using **PBAAA** as ligand.

Sample	Temperature Range (°C)	Mass Loss (%)	Decomposition Temperature (°C)	Residue
**PBAAA-Co 1:1_s**	25–200	11.2	893	10.6%
	201–450	77.6		
	450–893	0.6		
**PBAAA-Cu 2:1_s**	25–150	7	485	14.1%
	151–310	29.0		
	311–490	49.9		
**PBAAA-Cu 1:1_s**	25–150	6.5	478	18.5%
	151–320	29.3		
	321–475	45.7		
**PBAAA-Cu 1:2_s**	25–208	12.7	483	18.6%
	209–300	22.7		
	301–485	46.0		
**PBAAA-Zn 1:1_s**	25–150	7.5	520	14.4%
	151–420	28.6		
	421–520	49.5		

**Table 2:** TGA data for metal complexes of **PAAA**.

Sample	Temperature Range (°C)	Mass Loss (%)	Decomposition Temperature (°C)	Residue
**PAAA-Co 1:1_s**	25–130	15.0	455 (890)	18.5%
	131–245	8.5		
	246–455	56.8		
	455–490	1.2		
**PAAA-Cu 1:1_s**	25–127	4.5	495	26.0%
	128–220	9.9		
	221–320	11.0		
	321–370	16.7		
	371–495	31.9		
**PAAA-Zn 1:1_s**	25–198	8.1	545	17.7%
	151–545	74.2		

**Table 3:** TGA data for metal complexes obtained through mechanochemical synthesis.

Sample	Temperature Range (°C)	Mass Loss (%)	Decomposition Temperature (°C)	Residue
**PBAAA-Co 1h_m**	25–126	3.7	860	15.3%
	127–290	20.8		
	246–455	57.1		
	456–860	3.1		
**PBAAA-Cu 1h_m**	25–126	5.5	870	15.6%
	127–308	16.9		
	309–480	59.9		
	371–870	2.1		
**PBAAA-Cu 2h_m**	25–125	8.1	883	10.3%
	151–320	35.8		
	321–480	44.6		
	481–883	1.2		
**PBAAA-Cu 4h_m**	25–179	8.6	929	6.6%
	180–339	10.8		
	340–417	11.9		
	418–587	54.6		
	588–929	7.4		
**PBAAA-Zn 1h_m**	25–172	10.1	992	6.6%
	173–354	11.3		
	355–437	22.8		
	438–512	9.2		
	513–992	40.8		

**Table 4:** Parameters of the metal ions in the polymeric complexes. Ionic radius values (in Å) are sourced from [46].

Ligand	Counter Ion			
		Co^2+^	Cu^2+^	Zn^2+^
**PBAAA**	Ionic radius	0.74	0.65	0.6
	CN	6	5	4
**PAAA**	Ionic radius	0.74	0.57	0.6
	CN	6	4	4

**Table 5:** Parameters obtained by analyzing XPS Cu2p for **PBAAA-Cu 1:1_s** and **PAAA-Cu 1:1_s**.

Sample	Species	Peak	Binding Energy (eV)	Spin–Orbit Splitting (eV)	At Conc. (at%)
**PBAAA-Cu 1:1_s**	Cu^+^	3/2	932.3	20.8	17
		1/2	953.1		
	Cu^2+^	3/2	934.6	20.2	82
		1/2	954.8		
**PAAA-Cu 1:1_s**	Cu^2+^	3/2	934.2	19.9	100
		1/2	954.08		

## Data Availability

The original contributions presented in this study are included in the article/supplementary material. Further inquiries can be directed to the corresponding author(s).

## Supplementary Materials

The following supporting information can be downloaded at: https://www.mdpi.com/article/10.3390/polym17182511/s1, Figure S1: Powder XRD pattern of **PBAAA** with deconvoluted peaks for crystallinity degree determination; Figure S2: Powder XRD pattern of **PAAA** with deconvoluted peaks for crystallinity degree determination; Figure S3: UV-Vis monitoring of the synthesis of polymer ligands P2 from P1; Figure S4: FTIR spectra of Co (**1**), Cu (**2b**) and Zn (**3**) metal complexes with P1 ligand using 1:1 ratio, in the solution synthesis; Figure S5: FTIR spectra of Cu metal complexes with P1 ligand using different ratios between metal and ligand, using in solution synthesis; Figure S6: FTIR spectra of Co (**4**), Cu (**5**) and Zn (**6**) metal complexes with P2 ligand using 1:1 ratio, using in solution synthesis; Figure S7: FTIR spectra of Co (**7**), Cu (**8**) and Zn (**9**) metal complexes with P1 ligand using 1:1 ratio, using mechanochemistry synthesis; Figure S8: FTIR spectra of Cu metal complexes with P1 ligand using different times of reaction, using mechanochemistry synthesis; Figure S9: All derivatograms and thermogravimetric curves for metal complexes reported in this work.
