# Supplementary material for: Exploring the Properties of Organometallic Lactone-Containing Poly(benzofuran-co-arylacetic Acid): Traditional Synthesis Versus Mechanosynthesis
Source: Polymers (Basel). 2025 Sep 17;17(18):2511. doi: 10.3390/polym17182511 (PMC12473338; doi:10.3390/polym17182511)
Supplement: Supplementary file 1 [file polymers-17-02511-s001.zip › polymers-3837715-supplementary.pdf]

polymers

Supporting information for:

Exploring the Properties of Organometallic Lactone-containing Poly(benzofuran-co-arylacetic acid): Traditional Synthesis versus Mechanosynthesis

Teodora Radu [1], Alexandrina Nan [1]*, Monica Dan [1], Maria Miclăuş [1] and Natalia Terenti [1,*]

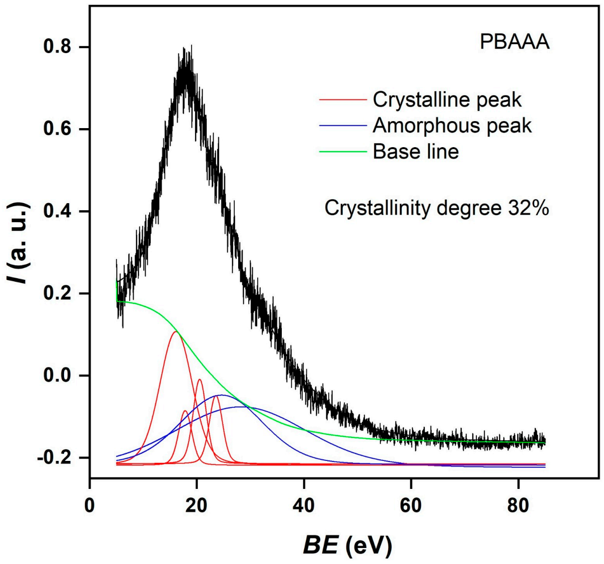

Figure S1: Powder XRD pattern of **PBAAA** with deconvoluted peaks for crystallinity degree determination.

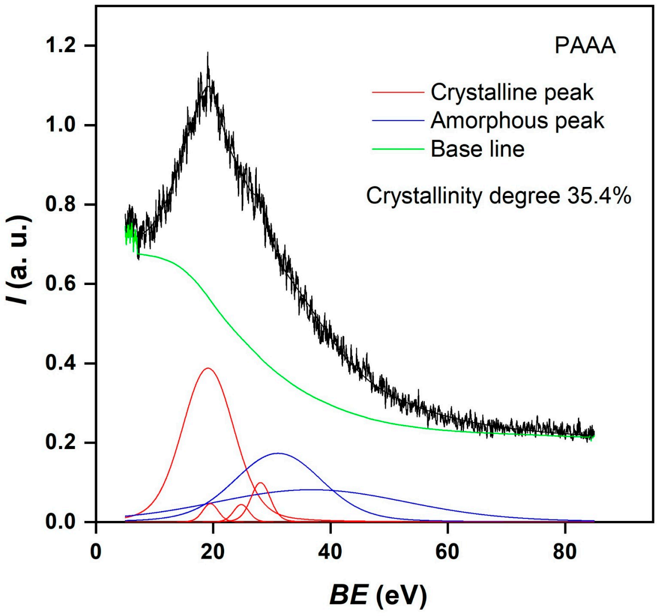

Figure S2: Powder XRD pattern of **PAAA** with deconvoluted peaks for crystallinity degree determination.

 https://doi.org/10.3390/xxxxx

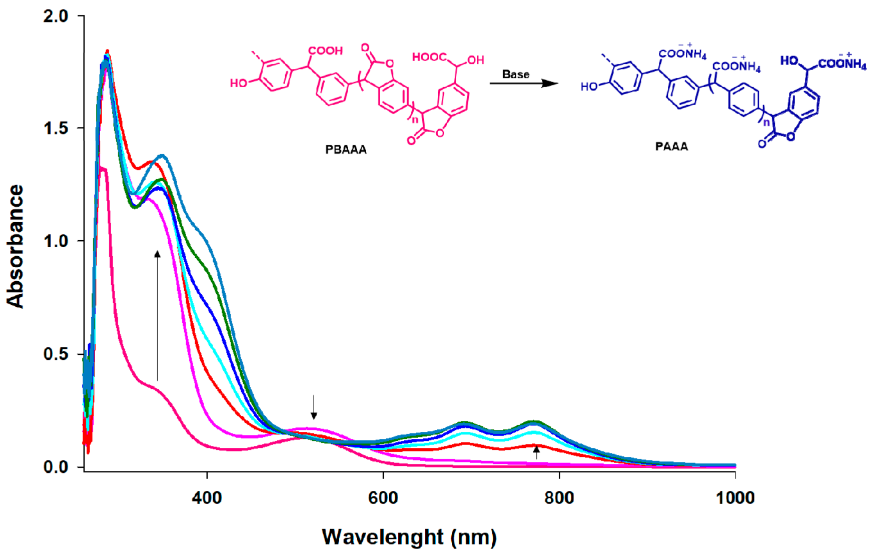

Figure S3: UV-Vis monitoring of the synthesis of polymer ligands **PAAA** from **PBAAA**.

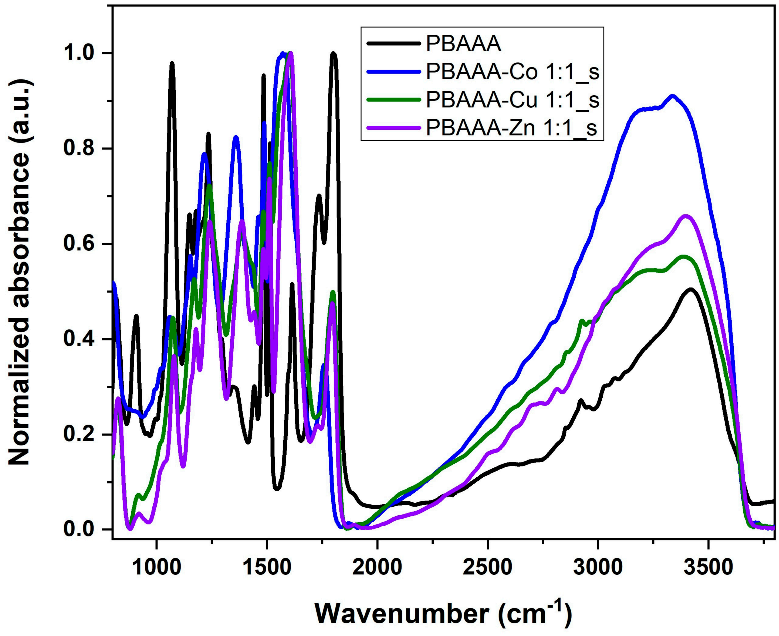

Figure S4: FTIR spectra of Co, Cu and Zn metal complexes with **PBAAA** ligand using 1: 1 ratio, in the solution synthesis

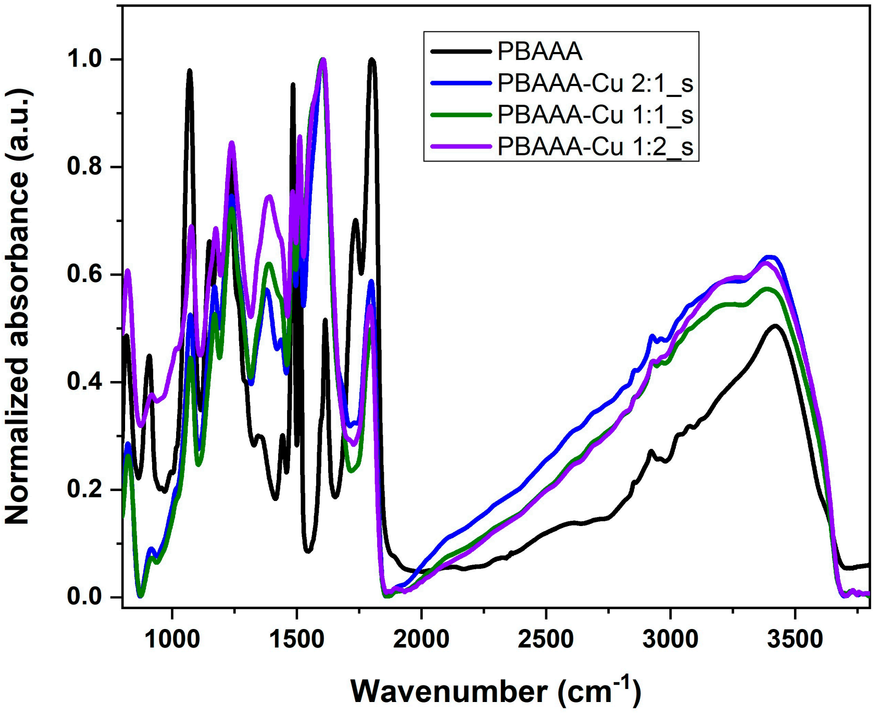

Figure S5: FTIR spectra of Cu metal complexes with **PBAAA** ligand using different ratios between metal and ligand, using in solution synthesis

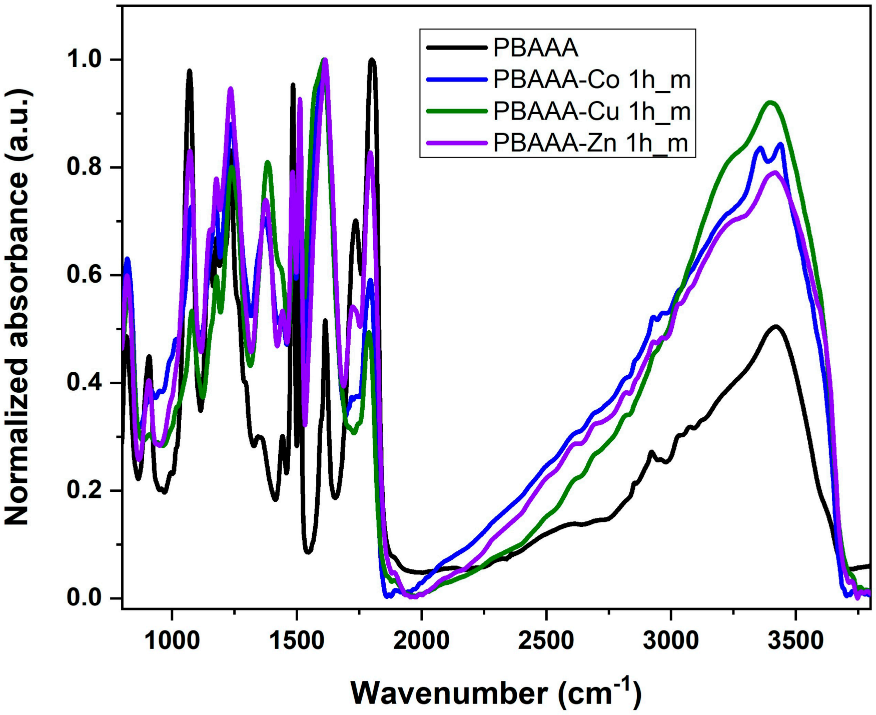

Figure S6: FTIR spectra of Co, Cu and Zn metal complexes with **PBAAA** ligand after 1h of reaction time, using mechanochemistry synthesis

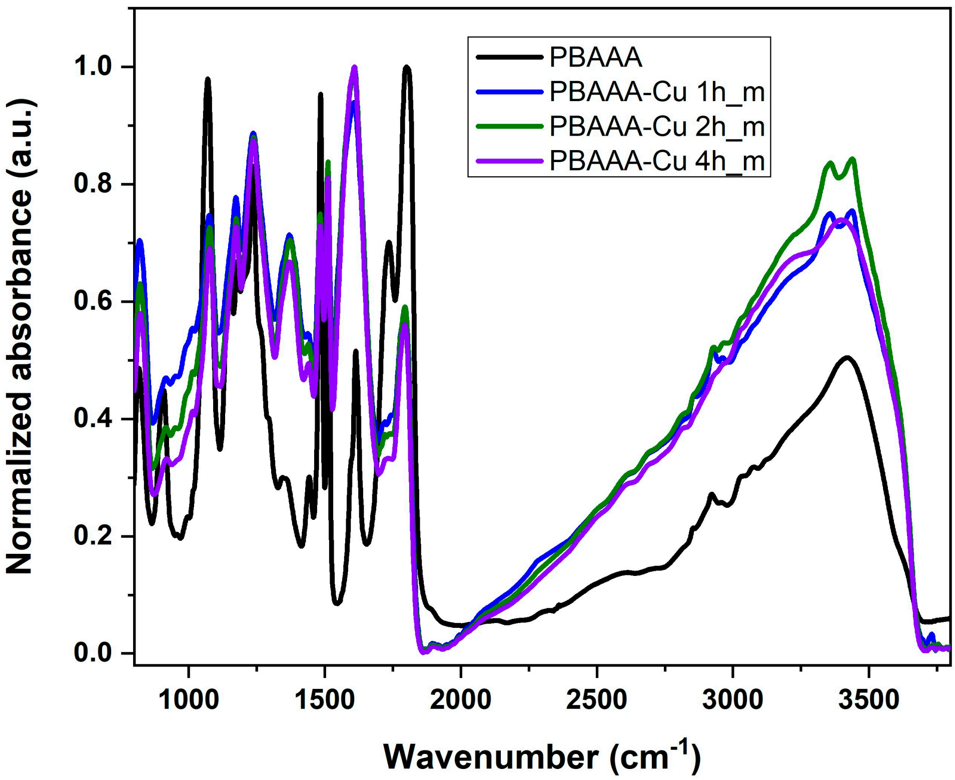

Figure S7: FTIR spectra of Cu metal complexes with **PBAAA** ligand using different reaction times, using mechanochemistry synthesis

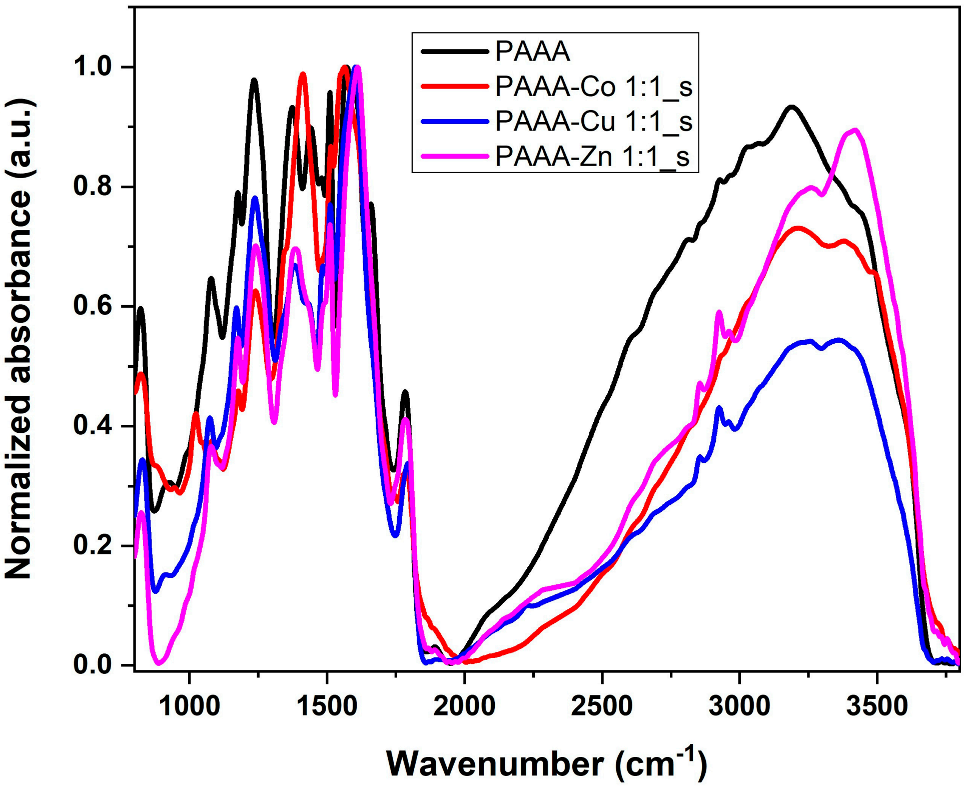

Figure S8: FTIR spectra of Co, Cu and Zn metal complexes with the **PAAA** ligand, used in solution synthesis

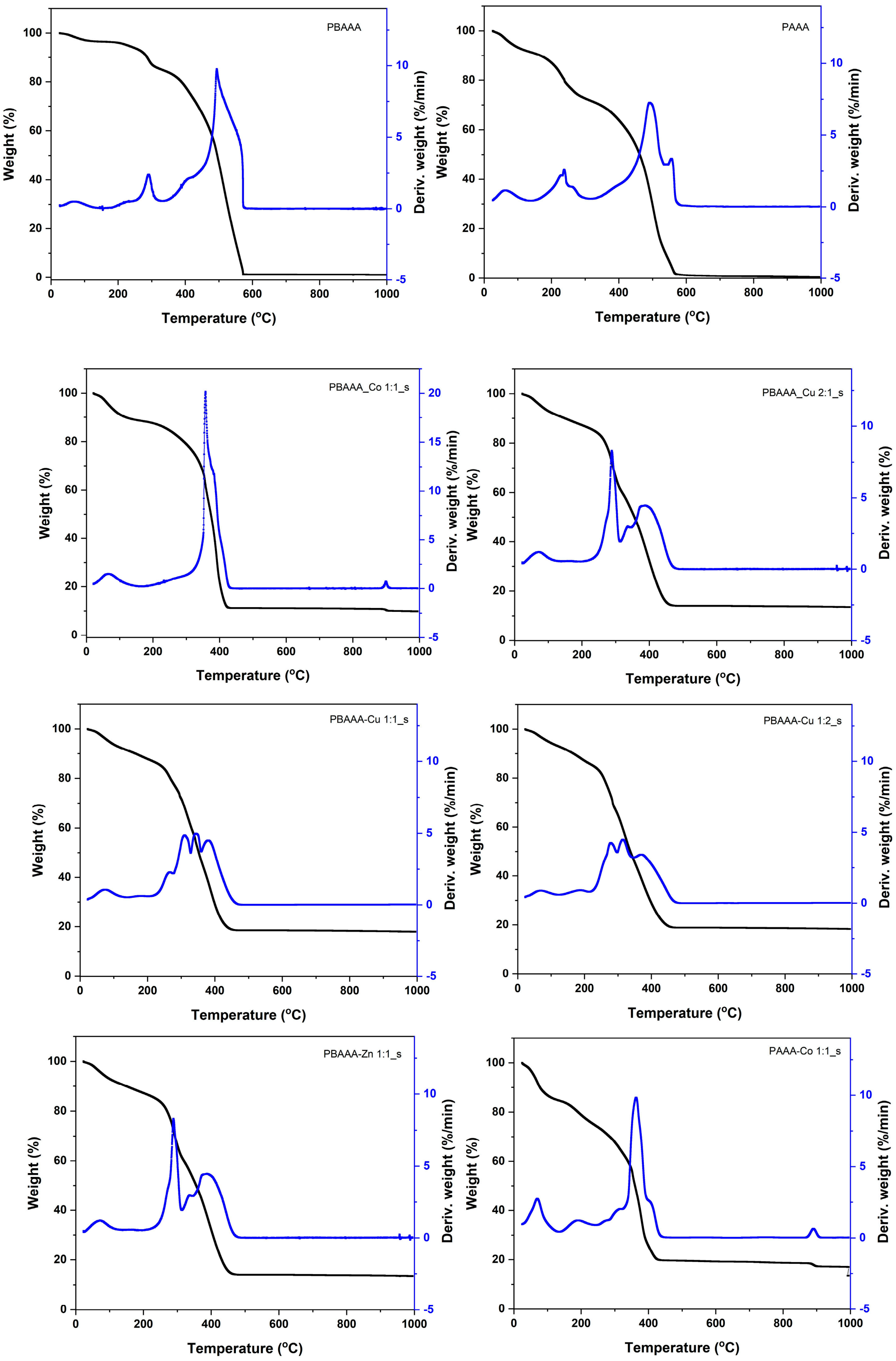
PBAAA
PAAA
PBAAA_Co 1:1_s
PBAAA_Cu 2:1_s
PBAAA-Cu 1:1_s
PBAAA-Cu 1:2_s
PBAAA-Zn 1:1_s
PAAA-Co 1:1_s
Weight (%)
Deriv. weight (%/min)
Deriv. weight (%)
Temperature (°C)

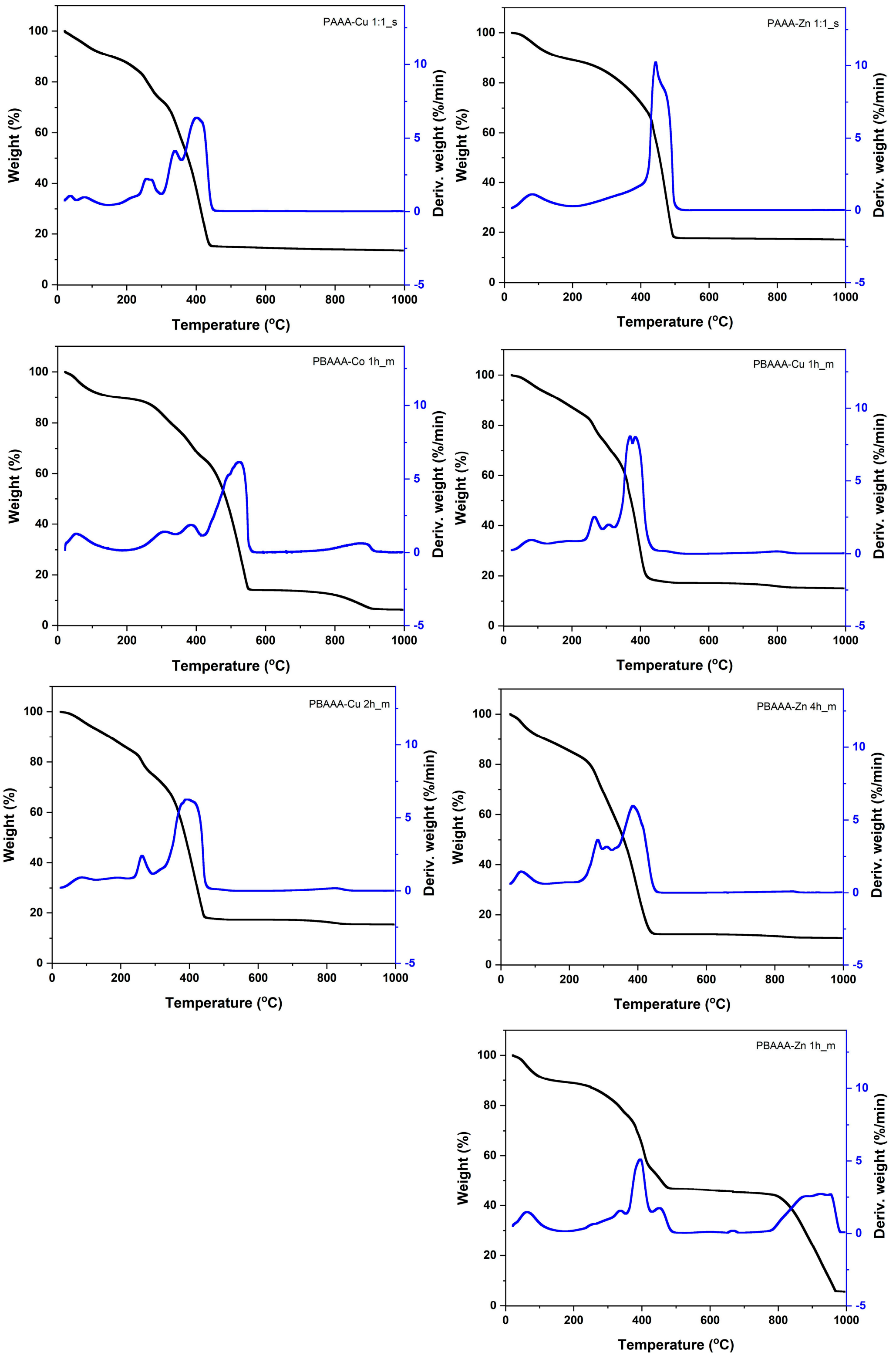

Figure S9: All derivatograms and thermogravimetric curves for metal complexes reported in this work.
